# Economic Hardships and Self-reported Deterioration of Physical and Mental Health Under the COVID-19 Pandemic: A Cross-sectional Study, 2020, Japan

**DOI:** 10.2188/jea.JE20210268

**Published:** 2022-04-05

**Authors:** Satomi Odani, Tomohiro Shinozaki, Kenji Shibuya, Takahiro Tabuchi

**Affiliations:** 1 Cancer Control Center, Osaka International Cancer Institute, Osaka, Japan; 2 Tokyo University of Science, Department of Information and Computer Technology, Tokyo, Japan; 3 Soma COVID Vaccination Medical Center, Fukushima, Japan; 4 The Tokyo Foundation for Policy Research, Tokyo, Japan

**Keywords:** mental health, self-rated health, health disparities, social determinants of health

## Abstract

**Background:**

Coronavirus disease 2019 (COVID-19) has disproportionately affected the most vulnerable populations. We assessed the prevalence and disparities of economic hardships and their impact on health deterioration in Japan.

**Methods:**

Data were obtained from a nation-wide, cross-sectional, internet-based, self-reported survey conducted during August–September, 2020 with individuals aged 15–79 years in Japan (*n* = 25,482). Economic hardships and changes in various physical and mental health status were measured using sample-weighted data. Adjusted prevalence ratios (APRs) were estimated to investigate the associations between economic hardships and health outcomes.

**Results:**

During April–September, 2020 in Japan, 25.0%, 9.6%, 7.9%, and 3.1% of the respondents experienced income loss, money shortage, financial anxiety and financial exploitation, respectively, with higher prevalence among workers (vs non-workers). Stratifying by sex and working status, income loss was associated with physical health deterioration (APRs ranged from 1.45–1.95), mental health deterioration (APRs ranged from 1.47–1.68), and having serious psychological distress (APRs ranged from 1.41–2.01) across all strata. Shortage of money and financial anxiety were also associated with increased likelihood of all adverse health outcomes assessed, regardless of whether the hardships were pre-existing or experienced first time. Among non-working individuals, financial exploitation was associated with physical health deterioration among males (APR 1.88) and mental health deterioration among both males (APR 1.80) and females (APR 2.23), while such associations were not observed among working individuals.

**Conclusions:**

During the early phase of the COVID-19 epidemic, COVID-19-related economic hardships were associated with physical and mental health deterioration in Japan, particularly among the vulnerable populations. Timely and prompt responses are warranted to mitigate both economic and health burdens.

## INTRODUCTION

The novel coronavirus disease 2019 (COVID-19) has posed a devastating socioeconomic and health burden on the society. Many countries have implemented public health responses, including lockdown, border control, mobility restriction, and suspension of businesses. Although they are effective to minimize infection, sudden standstill of the society has resulted in tremendous economic strain that has jeopardized the well-being of the public.^1^

COVID-19 has disproportionately affected the most vulnerable populations^2^^–^^4^; racial/ethnic minority, low-income, and young individuals have faced disproportionately high rates of job loss and income loss in many countries.^4^^–^^6^ Such economic hardships may lead to shortage of resources for housing, food, and medical and essential services, underscoring the urgent need to develop robust safety net programs to help those who experience the greatest harms. To support the livelihood of people under the COVID-19 pandemic, many countries and regions, such as the United States, Republic of Korea, and Hong Kong, introduced financial relief programs.^7^^–^^9^ The Japanese government also launched the Special Cash Payments program in April 2020 to provide one-time, across-the-board ¥100,000 ($930 in USD) relief.^10^^,^^11^ While a recent study has implied protective effects of the relief program for the health of workers,^12^ whether the relief has reached the individuals who bear the heaviest burden, including non-working individuals, remains understudied.

There also is a concern that the COVID-19-induced economic turmoil would widen the pre-existing health inequalities.^6^^,^^13^^,^^14^ An assessment of workers in six European countries demonstrates that economic hardships, such as income loss, job loss, and workload decrease during the pandemic were associated with increased mental health complaints.^15^ This relationship was more apparent among workers in a lower social gradient. Several other studies of working populations support this finding^16^^–^^18^; there exists a knowledge gap as to whether this is generalizable to other domains of health and other countries with diverse COVID-19 impact and different social systems. Filling this knowledge gap will enable us to provide important implications to design future interventions to mitigate health and social impacts of COVID-19, especially among the most vulnerable populations.

The aim of this study is to assess the levels and distribution of prevalence in economic hardships during the COVID-19 pandemic and their impact on health status. We examined three key components of economic hardship (income poverty, material deprivation, and subjective financial stress)^19^^,^^20^ along with two indicators of COVID-19-related economic hardships to obtain a more comprehensive understanding of personal or interpersonal experiences. We investigated the effects of both pre-existing economic hardships and those instantaneously induced during the COVID-19 pandemic. We additionally conducted stratified analyses by age, sex, and working status to examine whether the impact of economic hardships differed by these demographics.

## METHODS

### Internet survey

We obtained data from the Japan COVID-19 and Society Internet Survey (JACSIS), a nation-wide, cross-sectional, internet-based, self-reported survey of individuals aged 15 to 79 years. The JACSIS survey was administered by a private vendor, Rakuten Insight Inc., that had nearly 2.2 million qualified panelists in 2019.^21^ At the time of registration, the panelists were asked to provide their demographic information and a web-based informed consent. The questionnaires were distributed to 224,389 panelists selected by sex, age, and residing prefecture using simple random sampling. Data collection started on August 25^th^ and closed on September 30^th^, 2020, when the target sample size of 28,000 was met (response rate, 12.5%). There were no missing responses as all questions were required to be answered. We excluded 2,518 individuals who provided unnatural or inconsistent responses using the algorithm we developed,^22^ which resulted in the final sample size of 25,482 individuals. The study was reviewed and approved by the Research Ethics Committee of the Osaka International Cancer Institute (no. 20094-2).

### Variables

#### Economic hardships (exposure variables)

To assess the three major components of economic hardship (income poverty, material deprivation, and subjective financial stress),^19^^,^^20^^,^^23^ we utilized the following indicators that correspond to each of the components: income loss, shortage of money to buy things or pay bills, and anxiety about household budget (financial anxiety). Income loss was assessed with the question “When your income before the COVID-19 pandemic is taken as 100, how would you rate your income now? If your income has decreased by half, please rate as 50; if increased to double, please rate as 200.” Rating under 100 was defined as income loss. Shortage of money was assessed by asking respondents whether they had experienced shortage of money to buy or pay for items, such as rent/mortgage, food, medical/dental fees, tuition, and other necessities during April–September, 2020. Financial anxiety was assessed by asking respondents whether they had felt anxious about their household budget during April–September, 2020. For money shortage and financial anxiety, respondents were also asked whether they had experienced the hardships continuously before the COVID-19 pandemic or experienced first time since the pandemic took place. We considered the latter as a proxy of the COVID-19-induced economic hardship for descriptive analyses, while both pre-existing hardships and those instantaneously arose during the pandemic were employed to investigate their effects on health.

We further assessed two relevant measures of economic hardship, financial exploitation and non-receipt of the across-the-board relief, to obtain a nuanced understanding of personal or interpersonal experiences during the pandemic among various populations. Financial exploitation was assessed by asking respondents whether their saving or pension was used or taken by someone, including their family, without their permission during April–September, 2020. Non-receipt of the cash relief was assessed by asking respondents whether they had received the Special Cash Payment ($930 relief) during the COVID-19 pandemic by the time of data collection (April–September, 2020).

#### Deterioration and current state of physical/mental health (outcome variables)

As we were particularly interested in changes in health status during the COVID-19 pandemic, we assessed self-reported deterioration of physical and mental health as the primary outcomes. Respondents were asked “*How does your current physical/mental health state compare to that in or before January 2020?*” in separate questions. Responses “*Worsened very much/somewhat*” were defined as health deterioration (vs “*Improved very much/somewhat*”, “*Unchanged*” and “*I don’t know*”).

To provide a cross-sectional landscape of the health of the public under the COVID-19 pandemic, we also assessed two measures of current health status as the secondary outcomes. Respondents were asked to rate their general health status using a 5-point Likert scale. Responses were dichotomized as to be favorable (excellent/very good/good) or unfavorable (poor/fair). Presence of serious psychological distress (SPD) in the past 30 days was also assessed using the Kessler 6 (K6) scale: K6 scores ≥13 were defined as having SPD.^24^

#### Respondents’ characteristics (confounders)

Assessed characteristics included sex; age; education; past-year income level, classified using the latest national baseline income (ie, poverty line)^25^; marital status; employment status; and the number of COVID-19 cases in the residing area (cumulative number of cases during January 1^st^–August 25^th^, 2020, of each prefecture was divided into tertiles and used to classify areas of respondents’ residence).^26^ History of physical morbidities (cancer or malignant tumor, diabetes, asthma, bronchitis, chronic obstructive pulmonary disease, angina, myocardial infarction, and/or cerebral infarction) and mental illness (depression or any other mental disorder) was also assessed.

### Statistical analysis

To account for the potential selection bias of the internet-based sample, data were weighted using inverse probability weighting (IPW). We used a nationally representative population-based sample from the Comprehensive Survey of Living Conditions of People on Health and Welfare (CSLCPHW).^27^ Data from the two surveys (JACSIS and CSLCPHW) were combined and used for a logistic regression accounting for basic demographic characteristics such as sex, age, region, marital status, education, employment, and health status to estimate the probability of “being a respondent in the internet survey.” Further details regarding IPW are reported elsewhere.^22^^,^^28^

Adjusted prevalence ratios (APRs) were estimated through weighted multivariable-adjusted log-linear models fitted by the least-square method followed by robust variance estimation (also known as “modified Poisson” regression) to account for IPW and circumvent the specification of outcome distributions. First, we modeled the prevalence of economic hardships and health outcome measures as separate outcome (dependent) variables to investigate their possible predictors. We further compared the prevalence of health outcomes between presence versus absence of economic hardships. We fitted separate models for each of the combinations of an economic hardship variable and a health outcome variable within each of the population subgroups stratified by sex, age, and working status (working vs non-working). All analyses were conducted using R version 3.4.1 (R Foundation for Statistical Computing, Vienna, Austria).

## RESULTS

### Economic hardships during the COVID-19 pandemic

Demographic and socioeconomic characteristics of respondents are presented in eTable 1. Weighted analyses showed that, among all respondents, the prevalence of economic hardships during the COVID-19 pandemic was 25.0% (income loss), 9.6% (money shortage), 7.9% (financial exploitation), 3.1% (financial exploitation), and 13.6% (non-receipt of the relief) (Table 1). Working individuals had higher prevalence of income loss, money shortage, financial anxiety, and financial exploitation among both males and females (eTable 2 and eTable 3).

**Table 1.** Prevalence and correlates of economic hardships during the COVID-19 pandemic, 2020, Japan

	Respondent distribution	Income loss^a^		Money shortage^b^		Financial anxiety^c^		Financial exploitation^d^		Non-receipt of the cash relief^e^	
					
	*N* (%)	Prevalence (%)	APR (95% CI)	Prevalence (%)	APR (95% CI)	Prevalence (%)	APR (95% CI)	Prevalence(%)	APR (95% CI)	Prevalence (%)	APR (95% CI)
Overall	25,482 (100)	25.0	—	9.6	—	7.9	—	3.1	—	13.6	—
Sex											
Male	12,673 (49.7)	23.9	Ref.	11.4	Ref.	5.6	Ref.	5.0	Ref.	17.7	Ref.
Female	12,809 (50.3)	26.0	**1.28 (1.14–1.45)**	7.9	1.07 (0.78–1.46)	10.2	**2.02 (1.56–2.62)**	1.2	**0.45 (0.32–0.64)**	9.5	**0.58 (0.46–0.74)**
Age, years											
15–24	2,813 (10.9)	21.4	1.08 (0.71–1.65)	26.0	**4.42 (1.85–10.6)**	7.5	**2.06 (1.09–3.90)**	3.9	**5.14 (1.90–13.92)**	35.8	**5.11 (3.06–8.53)**
25–34	3,405 (13.2)	21.5	0.97 (0.72–1.29)	18.1	**2.46 (1.13–5.36)**	10.8	**2.38 (1.28–4.44)**	13.4	**16.04 (9.08–28.35)**	28.4	**4.11 (2.39–7.09)**
35–44	4,278 (16.9)	26.4	1.16 (0.92–1.45)	5.9	1.20 (0.58–2.47)	9.9	**2.00 (1.08–3.73)**	2.2	**4.94 (2.79–8.74)**	9.4	**1.94 (1.15–3.26)**
45–54	4,817 (18.9)	30.2	**1.36 (1.10–1.68)**	6.3	1.28 (0.65–2.52)	9.6	**1.99 (1.11–3.58)**	1.3	**2.98 (1.67–5.32)**	8.3	1.66 (0.99–2.76)
55–64	4,017 (15.4)	27.1	1.23 (0.99–1.50)	4.6	0.86 (0.46–1.60)	6.3	1.27 (0.72–2.25)	1.1	**2.40 (1.31–4.37)**	7.9	1.52 (0.90–2.59)
≥65	6,152 (24.8)	22.2	Ref.	6.1	Ref.	4.7	Ref.	0.4	Ref.	6.3	Ref.
Education											
Junior high/high school	8,291 (48.4)	23.6	0.90 (0.77–1.04)	6.8	0.68 (0.47–1.00)	7.8	1.09 (0.81–1.45)	2.6	**1.60 (1.14–2.25)**	11.8	0.97 (0.73–1.29)
Some college	5,387 (18.2)	26.0	0.89 (0.78–1.02)	7.1	0.79 (0.56–1.12)	10.3	1.11 (0.83–1.47)	2.0	**1.52 (1.04–2.21)**	9.3	0.92 (0.70–1.23)
College or higher	11,742 (33.4)	26.6	Ref.	15.3	Ref.	6.7	Ref.	4.4	Ref.	18.3	Ref.
Income level in the past year^f^											
≥Baseline income (≥twice the baseline)	12,585 (46.7)	28.6	Ref.	10.6	Ref.	7.8	Ref.	3.8	Ref.	12.5	Ref.
≥Baseline income (<twice the baseline)	5,702 (23.1)	29.6	1.10 (0.95–1.27)	9.4	1.10 (0.76–1.60)	9.4	1.34 (0.97–1.84)	1.4	0.62 (0.35–1.10)	9.1	0.89 (0.65–1.22)
<Baseline income	1,921 (8.3)	24.1	0.95 (0.78–1.16)	20.3	**1.71 (1.19–2.45)**	9.5	**1.46 (1.07–1.98)**	8.6	1.63 (0.93–2.85)	26.6	**1.61 (1.24–2.08)**
Indeterminate (did not answer)	5,274 (22)	12.8	**0.47 (0.37–0.58)**	3.8	**0.52 (0.37–0.73)**	5.7	**0.75 (0.58–0.97)**	1.4	0.79 (0.44–1.40)	15.6	**1.59 (1.31–1.93)**
Marital status											
Married	15,230 (63.2)	26.9	Ref.	6.4	Ref.	8.7	Ref.	1.4	Ref.	7.7	Ref.
Single	7,806 (23.7)	23.1	0.94 (0.78–1.13)	7.1	**0.57 (0.33–0.99)**	7.4	**0.65 (0.53–0.80)**	2.9	1.19 (0.89–1.58)	17.8	1.05 (0.89–1.24)
Divorced/widowed	2,446 (13.1)	19.0	**0.58 (0.41–0.82)**	29.8	**1.72 (1.11–2.67)**	4.6	**0.41 (0.28–0.61)**	11.8	**1.80 (1.14–2.84)**	34.0	**2.32 (1.72–3.14)**
Employment status											
Full-time employee	9,513 (34.9)	24.1	Ref.	8.3	Ref.	7.6	Ref.	3.6	Ref.	14.5	Ref.
Self-employed	1,645 (7.9)	47.1	**2.08 (1.70–2.54)**	17.6	1.58 (0.98–2.55)	11.8	**1.86 (1.16–2.98)**	7.4	1.15 (0.55–2.39)	12.1	0.71 (0.32–1.58)
Part-time employee, contractor	4,296 (19.1)	28.2	1.15 (0.99–1.34)	13.6	**1.48 (1.01–2.15)**	8.8	0.91 (0.73–1.12)	4.2	0.90 (0.49–1.65)	13.3	0.93 (0.71–1.22)
Non-working	10,028 (38.2)	19.6	0.88 (0.76–1.03)	7.2	1.18 (0.75–1.87)	6.9	0.86 (0.65–1.13)	1.2	0.81 (0.51–1.30)	13.1	1.17 (0.97–1.42)
Cumulative COVID-19 cases in residing area^g^											
1st tertile (1–12 cases per 100,000)	5,245 (33.8)	24.1	Ref.	6.5	Ref.	8.6	Ref.	2.0	Ref.	9.7	Ref.
2nd tertile (13–29 cases per 100,000)	5,116 (22.1)	23.6	0.98 (0.85–1.12)	7.4	1.00 (0.69–1.45)	7.1	0.82 (0.65–1.05)	1.6	0.76 (0.46–1.25)	12.2	1.09 (0.84–1.42)
3rd tertile (30–149 cases per 100,000)	15,121 (44.1)	26.4	1.08 (0.96–1.21)	13.2	1.10 (0.76–1.60)	7.7	0.95 (0.72–1.26)	4.7	1.07 (0.76–1.50)	17.2	**1.27 (1.07–1.51)**
History of physical condition^h^											
Never	17,840 (67.8)	24.5	Ref.	7.2	Ref.	7.7	Ref.	1.6	Ref.	12.4	Ref.
Past	4,566 (17.0)	23.0	0.93 (0.81–1.07)	9.2	1.19 (0.88–1.62)	9.3	1.21 (0.98–1.50)	4.3	**1.78 (1.30–2.43)**	11.6	1.04 (0.73–1.49)
Present	3,076 (15.2)	29.2	1.16 (0.94–1.42)	20.9	1.24 (0.65–2.36)	6.9	1.00 (0.66–1.51)	8.5	**2.46 (1.72–3.53)**	20.8	1.11 (0.77–1.59)
History of mental condition^h^											
Never	22,342 (85.1)	24.1	Ref.	6.7	Ref.	7.2	Ref.	1.5	Ref.	11.6	Ref.
Past	1,691 (7.2)	27.6	1.12 (0.92–1.35)	15.6	**1.81 (1.26–2.61)**	11.2	**1.37 (1.08–1.75)**	8.5	**2.30 (1.66–3.18)**	14.1	1.08 (0.70–1.67)
Present	1,449 (7.7)	32.8	**1.30 (1.00–1.70)**	35.9	1.56 (0.64–3.79)	12.2	1.86 (0.97–3.56)	15.7	**2.65 (1.75–4.02)**	35.1	1.20 (0.83–1.74)

APR, adjusted prevalence ratio; CI, confidence interval; COVID-19, coronavirus disease 2019; Ref, reference.

The prevalence and prevalence ratios were weighted to account for selection in an internet survey. Adjusted prevalence ratios and 95% confidence intervals were calculated using Poisson regression models adjusted for all the tabulation variables presented. Results in **bold type** indicate *P* < 0.05. See also eTable 1 for detailed definitions of economic hardship measures.

^a^Income loss was assessed by asking respondents whether their income had decreased compared to that before the COVID-19 pandemic.

^b^Money shortage to buy things or pay bills was defined as any problems to pay for items including rent/mortgage, food, medical/dental fees, tuition, and other necessities. Percentages that reported experiencing such problems for the first time during the COVID-19 pandemic were calculated.

^c^Financial anxiety was assessed by asking respondents whether they had felt anxious about the household budget during April–September 2020.

^d^Financial exploitation was defined as an event that respondents’ saving or pension was used or taken by someone including their family without their permission.

^e^Non-receipt of the cash relief ($930 across-the-board handout) was defined as a denial to the question asking whether respondents had received the relief by the time of survey.

^f^Past-year income level was classified using the 2018 national baseline income (poverty line).

^g^Cumulative COVID-19 cases during January 1^st^–August 25^th^, 2020 were calculated for each of the prefectures of respondents’ residence.

^h^History of illness was assessed with aggregate variables for physical illness (any cancer or malignant tumor, diabetes, asthma, bronchitis, chronic obstructive pulmonary disease, angina, myocardial infarction, and/or cerebral infarction) and mental illness (depression or any other mental disorder).

While females were more likely to report income loss (APR 1.28; 95% confidence interval [CI], 1.14–1.45) and financial anxiety (APR 2.02; 95% CI, 1.56–2.62), they were less likely to report financial exploitation (APR 0.45; 95% CI, 0.32–0.64) and non-receipt of the relief (APR 0.58; 95% CI, 0.46–0.74) compared to males (Table 1). Younger individuals were more likely to experience the assessed economic hardships, except income loss: the strongest association was seen for financial exploitation among individuals aged 25–34 years (APR 16.04; [95% CI, 9.08–28.35 vs the oldest group). Financial exploitation was also more likely among individuals with lower education (APR 1.60; 95% CI, 1.14–2.25 and APR 1.52; 95% CI, 1.04–2.21 among those with ≤high school education and some college education vs college graduates) and those with past or present physical chronic disease.

Compared to the most affluent group, individuals whose household past-year income was below the baseline had 1.71 (95% CI, 1.19–2.45), 1.46 (95% CI, 1.07–1.98), and 1.61 (95% CI, 1.24–2.08) times higher likelihood of experiencing money shortage, financial anxiety, and non-receipt of the relief, respectively. Divorced/widowed individuals were more likely to experience money shortage (APR 1.72; 95% CI, 1.11–2.67), financial exploitation (APR 1.80; 95% CI, 1.14–2.84) and non-receipt of the relief (APR 2.32; 95% CI, 1.72–3.14), while they had lower likelihood of experiencing income loss (APR 0.58; 95% CI, 0.41–0.82) and financial anxiety (APR 0.41; 95% CI, 0.28–0.61) compared to married individuals.

With full-time employees as the referent, employment status was significantly associated with income loss (APR 2.08; 95% CI, 1.70–2.54 among self-employed individuals), money shortage (APR 1.48; 95% CI, 1.01–2.15 among part-time workers/contractors), and financial anxiety (APR 1.86; 95% CI, 1.16–2.98 among self-employed individuals) during the COVID-19 pandemic. Respondents living in the areas with a higher number of COVID-19 infections were more likely to report non-receipt of the relief (APR 1.27; 95% CI, 1.07–1.51).

### Deterioration of physical/mental health and current state of health

Among all respondents, 15.5% and 18.5% reported their physical and mental health had worsened during the COVID-19 pandemic, respectively (Table 2). Current health status was rated as unfavorable by 13.5% of respondents. Prevalence of SPD was 10.0%, with a wide disparity between male workers and non-workers (13.9% vs 3.3%), while the difference was not as substantial between female workers and non-workers (9.5% vs 8.5%) (eTable 4 and eTable 5).

**Table 2.** Prevalence and correlates of deterioration of physical or mental health and current state of health during the COVID-19 pandemic, 2020, Japan

	Respondent distribution	Physical health deterioration^a^		Mental health deterioration^a^		Unfavorable general health status^b^		Serious psychological distress^c^	
				
	*N* (%)	Prevalence (%)	APR (95% CI)	Prevalence (%)	APR (95% CI)	Prevalence (%)	APR (95% CI)	Prevalence (%)	APR (95% CI)
Overall	25,482 (100)	15.5	—	18.5	—	13.5	—	10.0	—
Sex									
Male	12,673 (49.7)	15.5	Ref.	16.1	Ref.	14.6	Ref.	11.0	Ref.
Female	12,809 (50.3)	15.5	1.13 (0.91–1.41)	21.0	**1.35 (1.10–1.66)**	12.5	0.94 (0.71–1.24)	9.1	**1.28 (1.00–1.65)**
Age, years									
15–24	2,813 (10.9)	22.4	**1.72 (1.08–2.75)**	30.9	**2.49 (1.61–3.85)**	11.0	0.48 (0.23–1.01)	28.9	**5.31 (2.31–12.18)**
25–34	3,405 (13.2)	21.6	**1.86 (1.13–3.05)**	23.9	**1.97 (1.22–3.18)**	14.8	0.86 (0.42–1.75)	17.7	**3.29 (1.48–7.28)**
35–44	4,278 (16.9)	14.7	**1.52 (1.06–2.16)**	18.7	**1.65 (1.16–2.35)**	10.6	0.88 (0.59–1.31)	9.4	**2.45 (1.07–5.60)**
45–54	4,817 (18.9)	13.5	**1.41 (1.01–1.98)**	18.0	**1.65 (1.16–2.33)**	12.4	1.03 (0.72–1.49)	7.3	2.03 (0.91–4.53)
55–64	4,017 (15.4)	15.5	**1.51 (1.11–2.07)**	17.4	**1.58 (1.14–2.19)**	13.8	1.06 (0.78–1.43)	4.5	1.33 (0.60–2.96)
≥65	6,152 (24.8)	11.3	Ref.	11.2	Ref.	16.6	Ref.	3.6	Ref.
Education									
Junior high/high school	8,291 (48.4)	14.0	0.92 (0.73–1.17)	17.0	0.87 (0.70–1.07)	13.6	1.00 (0.73–1.37)	7.7	0.78 (0.58–1.06)
Some college	5,387 (18.2)	14.9	0.95 (0.76–1.18)	20.1	0.95 (0.78–1.15)	10.6	0.86 (0.64–1.17)	8.3	0.86 (0.65–1.14)
College or higher	11,742 (33.4)	17.7	Ref.	19.8	Ref.	14.8	Ref.	14.4	Ref.
Income level in the past year^d^									
≥Baseline income (≥twice the baseline)	12,585 (46.7)	15.9	Ref.	17.1	Ref.	11.9	Ref.	10.5	Ref.
≥Baseline income (<twice the baseline)	5,702 (23.1)	16.7	1.08 (0.81–1.43)	19.6	1.24 (0.99–1.55)	15.4	1.17 (0.84–1.63)	10.1	1.20 (0.82–1.76)
<Baseline income	1,921 (8.3)	21.9	1.12 (0.70–1.79)	24.1	1.34 (0.89–2.02)	23.2	1.37 (0.81–2.32)	16.6	**1.49 (1.04–2.14)**
Indeterminate (did not answer)	5,274 (22)	11.0	**0.71 (0.58–0.87)**	18.4	1.08 (0.88–1.33)	11.4	0.94 (0.71–1.26)	6.4	0.82 (0.61–1.10)
Marital status									
Married	15,230 (63.2)	13.4	Ref.	16.3	Ref.	12.1	Ref.	5.9	Ref.
Single	7,806 (23.7)	15.5	0.95 (0.79–1.14)	22.4	0.96 (0.82–1.12)	13.1	1.27 (0.93–1.72)	11.6	0.96 (0.77–1.21)
Divorced/widowed	2,446 (13.1)	25.7	1.25 (0.88–1.76)	22.0	0.93 (0.63–1.37)	21.3	1.29 (0.87–1.91)	27.0	**1.55 (1.01–2.47)**
Employment/occupation									
Full-time employee	9,513 (34.9)	14.3	Ref.	19.0	Ref.	10.3	Ref.	9.9	Ref.
Self-employed	1,645 (7.9)	19.6	1.27 (0.78–2.07)	15.3	0.82 (0.48–1.41)	14.2	0.98 (0.54–1.80)	21.6	1.68 (0.94–3.02)
Part-time employee, contractor	4,296 (19.1)	16.2	1.07 (0.77–1.48)	19.8	0.92 (0.71–1.20)	14.0	1.13 (0.70–1.81)	12.1	0.99 (0.75–1.31)
Unemployed (retired, student, domestic worker)	10,028 (38.2)	15.4	1.31 (0.94–1.81)	18.1	0.98 (0.74–1.29)	16.1	**1.55 (1.02–2.35)**	6.7	0.85 (0.61–1.20)
Residential area by cumulative number of COVID-19 cases^e^									
1st tertile (1–12 cases per 100,000)	5,245 (33.8)	13.8	Ref.	17.5	Ref.	13.4	Ref.	7.8	Ref.
2nd tertile (13–29 cases per 100,000)	5,116 (22.1)	15.9	1.13 (0.91–1.40)	19.8	1.15 (0.95–1.39)	12.7	0.93 (0.71–1.22)	7.1	0.85 (0.64–1.11)
3rd tertile (30–149 cases per 100,000)	15,121 (44.1)	16.6	1.02 (0.84–1.23)	18.7	1.02 (0.86–1.20)	14.0	0.94 (0.76–1.17)	13.2	1.01 (0.76–1.35)
History of physical condition^f^									
Never	17,840 (67.8)	13.6	Ref.	18	Ref.	10.2	Ref.	7.8	Ref.
Past	4,566 (17.0)	16	1.15 (0.95–1.40)	18.2	1.04 (0.89–1.20)	13.1	1.20 (0.96–1.50)	10.7	1.20 (0.94–1.55)
Present	3,076 (15.2)	23.6	1.26 (0.96–1.65)	21.4	1.06 (0.77–1.45)	28.8	**2.04 (1.51–2.76)**	19.4	0.78 (0.41–1.50)
History of mental condition^f^									
Never	22,342 (85.1)	12.6	Ref.	15.9	Ref.	11	Ref.	5.6	Ref.
Past	1,691 (7.2)	20.6	**1.48 (1.13–1.92)**	25.5	**1.56 (1.28–1.91)**	15.4	**1.37 (1.03–1.80)**	20.1	**2.79 (2.08–3.75)**
Present	1,449 (7.7)	42.7	**2.58 (1.84–3.61)**	41.2	**2.30 (1.68–3.16)**	39.8	**3.07 (2.08–4.53)**	49.3	**5.02 (3.18–7.90)**

APR, adjusted prevalence ratio; CI, confidence interval; COVID-19, coronavirus disease 2019; Ref, reference.

The prevalence and prevalence ratios were weighted to account for selection in an internet survey. Adjusted prevalence ratios and 95% confidence intervals were calculated using Poisson regression models adjusted for all the tabulation variables presented. Results in **bold type** indicate *P* < 0.05.

^a^Deterioration of health were self-reported by asking respondents whether their state of health had worsened or improved compared to that in or during January 2020.

^b^Unfavorable general health status was defined as responses “poor” or “fair” to the question about respondents’ current health status using a 5-point Likert scale.

^c^Presence of serious psychological distress was defined as the score ≥13 on the Kessler (K6) scale.

^d^Past-year income level was classified using the latest national baseline income (poverty line).

^e^Cumulative COVID-19 cases during January 1^st^–August 25^th^, 2020 were calculated for each of the prefectures of respondents’ residence.

^f^History of illness was assessed with aggregate variables for physical illness (any cancer or malignant tumor, diabetes, asthma, bronchitis, chronic obstructive pulmonary disease, angina, myocardial infarction, and/or cerebral infarction) and mental illness (depression or any other mental disorder).

Females had higher likelihood of mental health deterioration (APR 1.35; 95% CI, 1.10–1.66) and reporting SPD (APR 1.28; 95% CI, 1.00–1.65) than males during the COVID-19 pandemic. Younger individuals were more likely to report deterioration of physical and mental health; prevalence of SPD was also higher among young individuals with the strongest association among those aged 15–24 years (APR 5.31; 95% CI, 2.31–12.18). SPD was also more often reported by individuals whose past-year income was below the baseline (APR 1.49; 95% CI, 1.04–2.14) and the divorced/widowed (APR 1.55; 95% CI, 1.01–2.47).

Compared to individuals without a mental illness history, those with past or present mental illness were more likely to report adverse health outcomes, with stronger associations among those currently having the illness (APR 2.58; 95% CI, 1.84–3.36 and APR 2.30; 95% CI, 1.68–3.16 for deterioration of physical and mental health, respectively, and APR 3.07; 95% CI, 2.08–4.53 and APR 5.02; 95% CI, 3.18–7.90 for unfavorable current health and SPD, respectively).

### Associations between economic hardships and physical/mental health during the COVID-19 pandemic

**Figure 1.**
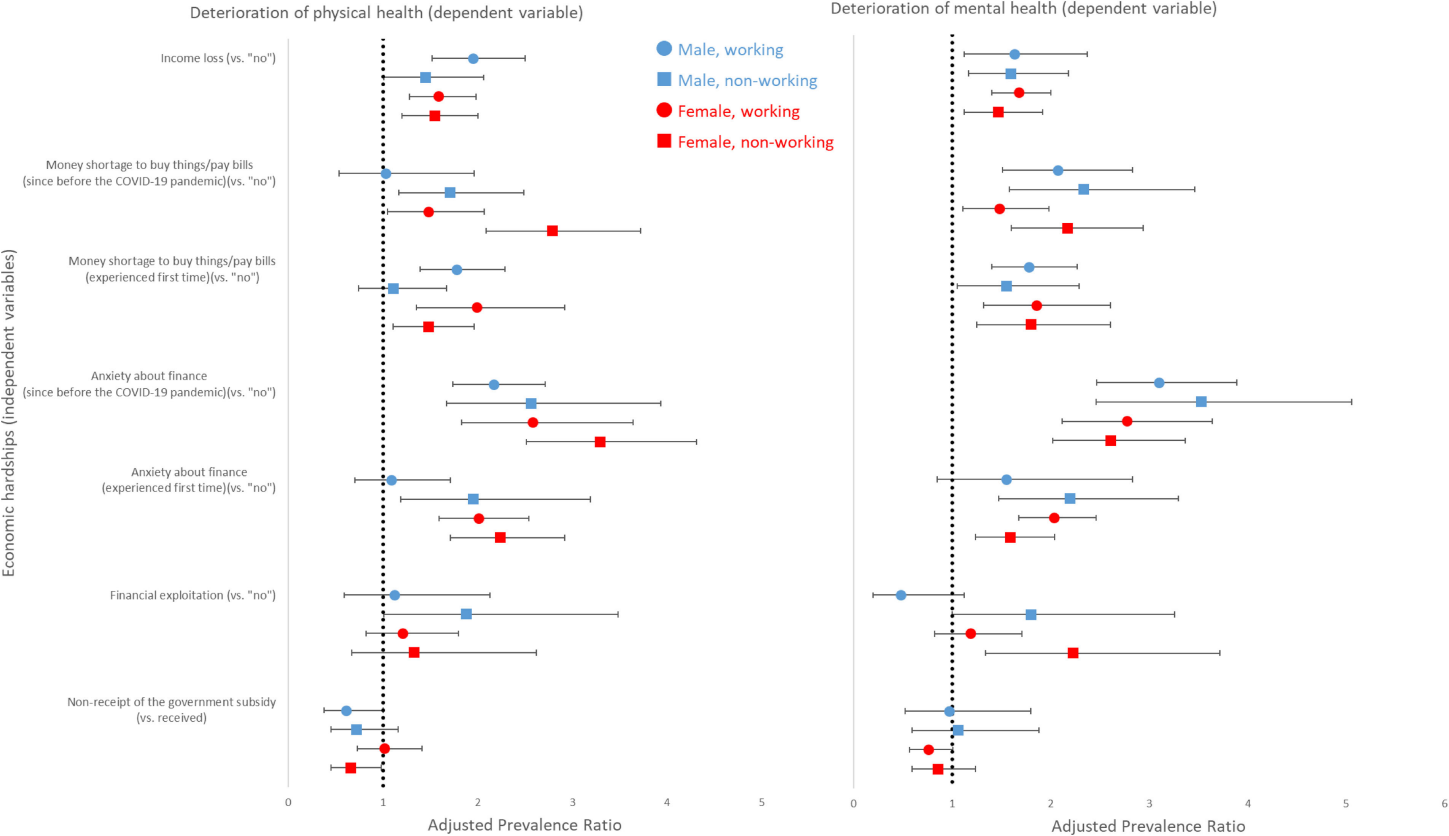
Associations between economic hardships and health deterioration during the COVID-19 pandemic, 2020, Japan. *N* = 9,008 (male, working); 3,665 (male, non-working); 6,446 (female, working); 6,363 (female, non-working). Gender- and working status-specific adjusted prevalence ratios (95% confidence intervals) estimated through weighted multivariable-adjusted log-linear models followed by robust variance estimation controlling for age, education, past-year income level, marital status, cumulative COVID-19 cases in residing area, and history of physical and mental illness. Working status was categorized as to be working (full-time employee, self-employed, part-time/contractor) or non-working (retired, student, domestic worker, unemployed). Deterioration of health was self-reported by asking respondents whether their state of health had worsened compared to that in or during January 2020. COVID-19, coronavirus disease 2019.

After adjusting for confounders, respondents who experienced income loss had 1.45–1.95 times higher likelihood of physical health deterioration and 1.47–1.68 times higher likelihood of mental health deterioration during the COVID-19 pandemic within all sex-work strata (Figure 1). APRs and CIs for overall and each sex-work strata are presented in eTable 6. Money shortage was also a significant predictor of health deterioration, regardless of whether the problem was pre-existing or not: the strongest associations were seen for physical health deterioration among non-working females who experienced money shortage for the first time during the COVID-19 pandemic (APR 2.79; 95% CI, 2.09–3.72) and for mental health deterioration among non-working males who experienced money shortage for the first time (APR 2.34; 95% CI, 1.58–3.46).

Similarly, non-working females and non-working males who reported experiencing financial anxiety for the first time during the COVID-19 pandemic had the highest likelihood of physical health deterioration (APR 3.29; 2.51–4.31) and mental health deterioration (APR 3.53; 95% CI, 2.46–5.06), respectively. While financial exploitation was not significantly associated with health deterioration in working populations, it significantly predicted physical health deterioration among non-working males (APR 1.88; 95% CI, 1.01–3.48) and mental health deterioration among non-working males (APR 1.80; 95% CI, 1.00–3.26) and non-working females (APR 2.23; 95% CI, 1.34–3.72). Non-receipt of the cash relief was associated with lower likelihood of physical health deterioration among non-working females (APR 0.66; 0.45–0.99).

**Figure 2.**
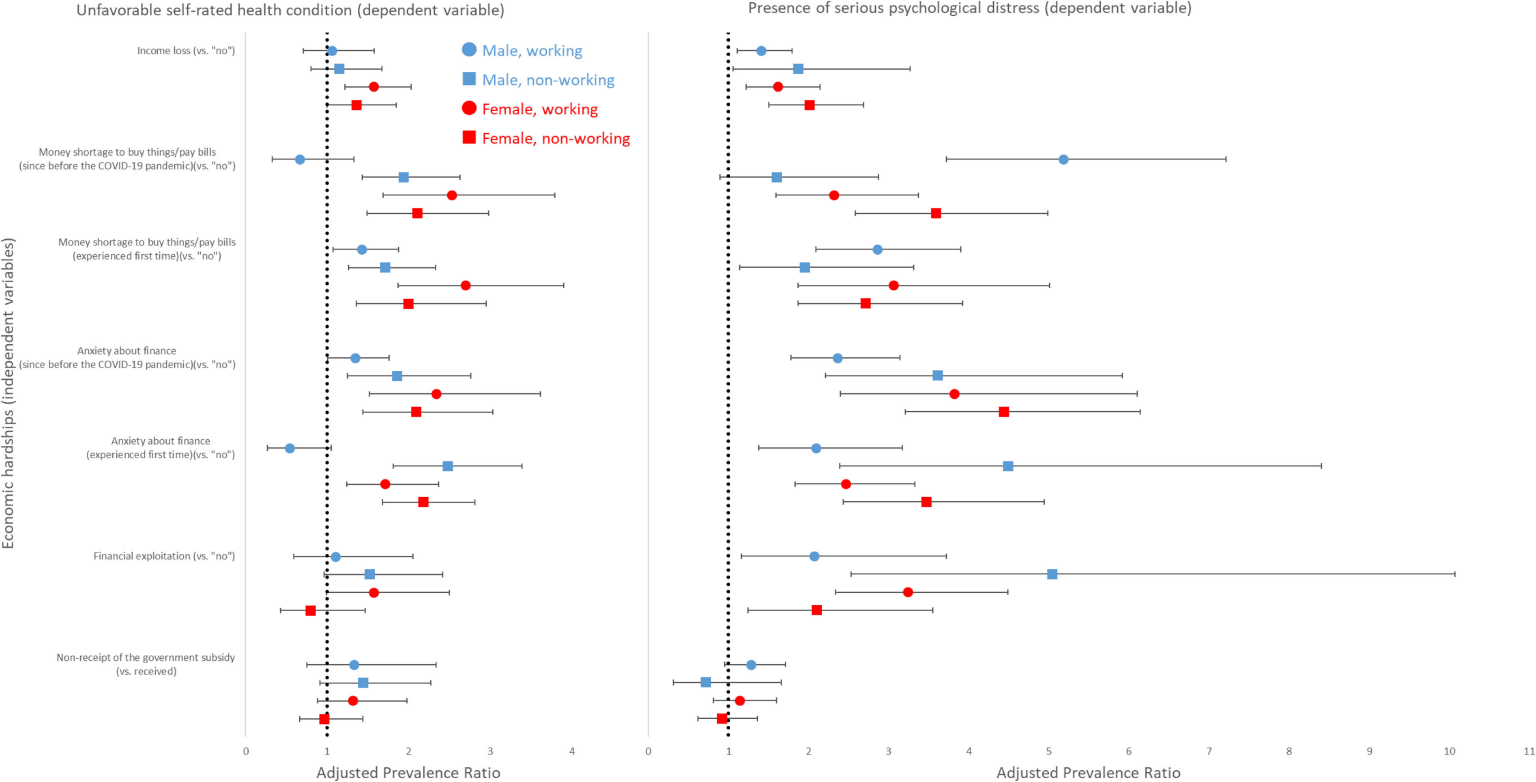
Associations between economic hardships and current state of health during the COVID-19 pandemic, 2020, Japan. *N* = 9,008 (male, working); 3,665 (male, non-working); 6,446 (female, working); 6,363 (female, non-working). Gender- and working status-specific adjusted prevalence ratios (95% confidence intervals) estimated through weighted multivariable-adjusted log-linear models followed by robust variance estimation controlling for age, education, past-year income level, marital status, cumulative COVID-19 cases in residing area, and history of physical and mental illness. Working status was categorized as to be working (full-time employee, self-employed, part-time/contractor) or non-working (retired, student, domestic worker, unemployed). Current health condition was defined as to be unfavorable (poor/fair) or favorable (excellent, very good, good) using a 5-point Likers scale. Presence of serious psychological distress was defined as scores ≥13 using the Kessler-6 scale. COVID-19, coronavirus disease 2019.

Figure 2 and eTable 6 present associations between economic hardships and current state of health. Consistent to the findings in Figure 1, income loss, money shortage, and financial anxiety were significantly associated with unfavorable health condition and presence of SPD within most of sex-work strata. Notably, working males who experienced money shortage for the first time during the COVID-19 pandemic were 5.18 (95% CI, 3.72–7.21) times more likely to have SPD compared to those who did not report money shortage. Financial anxiety was associated with increased likelihood of both unfavorable current health and SPD, with generally stronger associations in non-working populations. Financial exploitation also predicted the presence of SPD with the strongest association among non-working males (APR 5.04; 95% CI, 2.53–10.07). eTable 7 presents stratified APRs by age (<65 vs ≥65 years) to investigate whether the impact of economic hardships differs for younger adults versus older adults based on the assumption that working status had different implications for these two age groups (ie, older adults were mostly retired and non-working). While the strengths of associations differed, the directions of associations were generally consistent between age groups.

## DISCUSSION

Economic hardships—income loss, shortage of money, and financial anxiety—were independently associated with increased likelihood of physical and mental health deterioration, being in unfavorable health conditions, and having SPD, regardless of whether such economic hardships were pre-existing or arose during the COVID-19 pandemic. Financial exploitation was associated with deterioration of physical and mental health among only non-working individuals. Our findings shed light on the vulnerability of certain populations and underscore the importance of timely and targeted interventions to help mitigate the detrimental impact of economic hardships.

We found that one in four individuals in Japan experienced income loss during the COVID-19 pandemic, with higher likelihood among females: more females reported financial anxiety than males. This can be partially attributable to the facts that more women in Japan serve in the industries, such as service, retail, or travel, which were severely damaged by the COVID-19 pandemic,^29^ and that women faced drastic changes in their employment status and reduced working hours more often than their male counterparts.^30^ Although there were no systematic patterns in disparities in income loss across age and past-year income, younger and less affluent individuals were more likely to experience money shortage and financial anxiety during the COVID-19 pandemic. Furthermore, divorced or widowed individuals were nearly twice as likely to experience money shortage, despite their relatively low likelihood of income loss. These findings indicate that income loss during the COVID-19 pandemic have exaggerated the pre-existing economic inequalities by burdening the finance of various populations, and consequently, vulnerable individuals had to face material deprivation and financial anxiety.

Our analysis of financial exploitation and non-receipt of the cash relief provides important implications regarding personal or interpersonal experiences, especially those of non-working populations. We found that 13.6% of all respondents had not received the relief at the time of survey. As of August 27^th^, 2020, when our data collection was taking place, Japan’s Ministry of Internal Affairs and Communications reported that the total program spending reached 12.59 trillion Japanese yen ($110 billion in United States dollars), which had seemingly achieved 98.9% coverage.^10^ It should be noted, however, that the relief program was administered on a household basis that allowed only the head of each household to file for and receive the relief on behalf of all household members. Therefore, the gap in non-receipt percentage between our finding (13.6%) and the official estimate (1.1%) might be partially due to stagnated distribution within each household.

We further found similar patterns in non-receipt of the cash relief and financial exploitation: both were more common among male, younger, and divorced or widowed individuals. Due to the household-based administration, receipt of the cash relief could be affected by the power relationship within each household. It should also be noted that individuals with a history of physical and mental illness were more likely to experience financial exploitation, indicating that their financial autonomy may have been limited under the pandemic. These facts underscore the need to evaluate the COVID-19-related support programs from the public perspective to inform future interventions and maximize the reach to disadvantaged individuals.

Japan marked a drastic increase in suicide incidence since July 2020, with the most prominent surge among young females.^30^^,^^31^ Recent assessments of Japanese workers have revealed a synchronized increase in psychological distress among younger females.^16^^,^^17^ Our study adds up to this evidence base by identifying the populations at higher risk of SPD and mental health deterioration including poorer individuals, divorced or widowed individuals, and those with a history of mental illness. We also found that individuals with a history of past or present mental illness were more likely to report physical health deterioration, possibly reflecting their limited resilience to cope with the physical or psychological stress imposed during the COVID-19 pandemic. Furthermore, exposure to financial exploitation was significantly associated with deterioration of physical and mental health among only non-working individuals, highlighting their vulnerability to the detrimental effects of such interpersonal experiences. Given that non-working population consists of the retired, students, and domestic workers, who might often lack social connectedness during or in the aftermath of the COVID-19 crisis, targeted interventions, such as remote counseling services and provision of information resources for individuals who seek help, are warranted.

Although we cannot infer causality from our cross-sectional data, our findings would suggest the bidirectional association between economic hardships and adverse health outcomes. Deterioration of physical and mental health was significantly higher among individuals who had been deprived of money or having financial anxiety since before the COVID-19 pandemic, suggesting that health outcomes were preceded by economic hardships. The opposite trajectory would also exist, given that individuals with a history of past morbidities were more likely to undergo economic hardships that arose during the pandemic. To address this bidirectional aspect of detrimental effects, implementation of social support programs to target the populations with heavier health burdens, in combination with safety net programs to mitigate the COVID-19-induced economic hardships, is necessary to prevent socioeconomic and health disparities from widening.

There are several limitations in the present study. First, due to the cross-sectional nature of the study, we were unable to establish the exact chronological mechanism of the association between exposure to economic hardships and health outcomes. Second, the self-reported nature of the survey might have resulted in misreport even after filtering out invalid responses. Third, as the study sample was collected through the internet-based recruitment, our findings may not be generalizable to the population with limited access or literacy to the internet. Nevertheless, such selection bias is considered to be minimal, as we used weighted data to address the differences in socioeconomic and demographic characteristics between respondents of the current internet survey and the nationally representative survey. Lastly, the limited sample size prevented us from nuanced analysis of economic hardships and health impacts that might have been experienced differently by type of industries that respondents belonged to.

### Conclusion

During the COVID-19 pandemic, three components of economic hardship—income loss, shortage of money, and financial anxiety—were independently associated with deterioration of physical and mental health, unfavorable current general health condition, and presence of SPD. Financial exploitation predicted mental health deterioration among non-working populations. Timely and targeted interventions, in combination with detailed assessment of the health of the at-risk populations, are warranted to prevent both the socioeconomic and health disparities from widening under the COVID-19 crisis.

## APPENDIX A. SUPPLEMENTARY DATA

eTable 1. Demographic and socioeconomic characteristics of respondents, 2020, Japan

eTable 2. Prevalence and correlates of economic hardships experienced during the COVID-19 pandemic among males by working status, 2020, Japan

eTable 3. Prevalence and correlates of economic hardships experienced during the COVID-19 pandemic among females by working status, 2020, Japan

eTable 4. Prevalence and correlates of deterioration of physical or mental health and current state of health during the COVID-19 pandemic among males by working status, 2020, Japan

eTable 5. Prevalence and correlates of deterioration of physical or mental health and current state of health during the COVID-19 pandemic among females by working status, 2020, Japan

eTable 6. Associations between economic hardships and self-reported health during the COVID-19 pandemic, 2020, Japan

eTable 7. Associations between economic hardships and self-reported health during the COVID-19 pandemic by age (<65 vs. ≥65 years), 2020, Japan
