# Supplementary material for: Economic Hardships and Self-reported Deterioration of Physical and Mental Health Under the COVID-19 Pandemic: A Cross-sectional Study, 2020, Japan
Source: J Epidemiol. 2022 Apr 5;32(4):195–203. doi: 10.2188/jea.JE20210268 (PMC8918622; doi:10.2188/jea.JE20210268)
Supplement: Supplementary file 1 [file je-32-195-s001.pdf]

**eTable 1.** Demographic and socioeconomic characteristics of respondents, 2020, Japan

| | Respondent distribution |
|---|---|
| | N (%) |
| Overall | 25,482 (100) |
| Sex | |
| Male | 12,673 (49.7) |
| Female | 12,809 (50.3) |
| Age, years | |
| 15–24 | 2,813 (10.9) |
| 25–34 | 3,405 (13.2) |
| 35–44 | 4,278 (16.9) |
| 45–54 | 4,817 (18.9) |
| 55–64 | 4,017 (15.4) |
| ≥65 | 6,152 (24.8) |
| Education | |
| Junior high/high school | 8,291 (48.4) |
| Some college | 5,387 (18.2) |
| College or higher | 11,742 (33.4) |
| Income level in the past year[a] | |
| ≥Baseline income (≥twice the baseline) | 12,585 (46.7) |
| ≥Baseline income (<twice the baseline) | 5,702 (23.1) |
| <Baseline income | 1,921 (8.3) |
| Indeterminate (did not answer) | 5,274 (22) |
| Marital status | |
| Married | 15,230 (63.2) |
| Single | 7,806 (23.7) |
| Divorced/widowed | 2,446 (13.1) |
| Employment status | |
| Full-time employee | 9,513 (34.9) |
| Self-employed | 1,645 (7.9) |
| Part-time employee, contractor | 4,296 (19.1) |
| Non-working | 10,028 (38.2) |
| Cumulative COVID-19 cases in residing area[b] | |
| 1st tertile (1–12 cases per 100,000) | 5,245 (33.8) |
| 2nd tertile (13–29 cases per 100,000) | 5,116 (22.1) |
| 3rd tertile (30–149 cases per 100,000) | 15,121 (44.1) |
| History of physical condition[c] | |
| Never | 17,840 (67.8) |
| Past | 4,566 (17.0) |
| Present | 3,076 (15.2) |
| History of mental condition[c] | |
| Never | 22,342 (85.1) |
| Past | 1,691 (7.2) |
| Present | 1,449 (7.7) |

COVID-19, coronavirus 2019.

Percentages were weighted to account for selection in an internet survey.

[a] Past-year income level was classified using the 2018 national baseline income (poverty line).

[b] Cumulative COVID-19 cases during January 1st–August 25th, 2020 were calculated for each of the prefectures of respondents' residence.

[c] History of illness was assessed with aggregate variables for physical illness (any cancer or malignant tumor, diabetes, asthma, bronchitis, chronic obstructive pulmonary disorder, angina, myocardial infarction, and/or cerebral infarction) and mental illness (depression or any other mental disorder).

**eTable 2.** Prevalence and correlates of economic hardships experienced during the COVID-19 pandemic among males by working status, 2020, Japan

| | Income loss[a] | | Money shortage[b] | | Financial anxiety[v] | | Financial exploitation[d] | | Non-receipt of the cash relief[e] | |
|---|---|---|---|---|---|---|---|---|---|---|
| | Working (N=9,008) Prevalence (%) | Non-working (N=3,665) Prevalence (%) | Working (N=9,008) Prevalence (%) | Non-working (N=3,665) Prevalence (%) | Working (N=9,008) Prevalence (%) | Non-working (N=3,665) Prevalence (%) | Working (N=9,008) Prevalence (%) | Non-working (N=3,665) Prevalence (%) | Working (N=9,008) Prevalence (%) | Non-working (N=3,665) Prevalence (%) |
| Male overall | 27.5 | 14.4 | 12.7 | 8.0 | 6.0 | 4.3 | 6.2 | 1.8 | 17.7 | 17.8 |
| Age, years | | | | | | | | | | |
| 15–24 | 23.8 | 18.6 | 75.0 | 8.6 | 2.4 | 8.0 | 1.7 | 7.9 | 59.7 | 42.3 |
| 25–34 | 18.2 | 5.6 | 21.2 | 61.4 | 6.8 | 2.8 | 27.7 | - | 41.6 | 74.2 |
| 35–44 | 26.7 | 22.5 | 4.4 | 6.2 | 6.9 | 5.0 | 2.7 | 3.9 | 10.5 | 22.7 |
| 45–54 | 31.7 | 29.9 | 5.5 | 7.1 | 7.7 | 7.0 | 1.5 | - | 8.3 | 16.7 |
| 55–64 | 30.8 | 22.1 | 4.1 | 2.4 | 4.0 | 6.3 | 1.9 | - | 8.9 | 11.9 |
| ≥65 | 30.9 | 11.2 | 4.0 | 3.6 | 5.2 | 2.9 | 1.3 | 0.4 | 3.1 | 6.3 |
| Education | | | | | | | | | | |
| Junior high/high school | 29.2 | 13.4 | 7.8 | 4.1 | 6.3 | 4.5 | 5.0 | 2.0 | 11.6 | 16.0 |
| Some college | 28.1 | 18.3 | 5.5 | 5.7 | 8.6 | 2.3 | 3.4 | 1.5 | 11.6 | 17.6 |
| College or higher | 26.0 | 14.7 | 20.0 | 19.5 | 5.1 | 3.6 | 8.3 | 1.3 | 25.7 | 22.4 |
| Income level in the past year[f] | | | | | | | | | | |
| ≥Baseline income (≥twice the baseline) | 27.5 | 18.6 | 12.7 | 6.3 | 5.5 | 5.5 | 6.2 | 3.5 | 17.8 | 9.5 |
| ≥Baseline income (<twice the baseline) | 33.1 | 18.5 | 14.2 | 3.3 | 9.0 | 3.5 | 2.8 | 0.3 | 14.2 | 7.2 |
| <Baseline income | 32.3 | 11.2 | 33.3 | 22.5 | 7.4 | 3.5 | 29.3 | 3.1 | 35.4 | 38.3 |
| Indeterminate (did not answer) | 17.6 | 6.6 | 2.2 | 4.3 | 4.0 | 4.6 | 1.2 | 1.2 | 14.6 | 24.4 |
| Marital status | | | | | | | | | | |
| Married | 29.9 | 13.9 | 5.1 | 3.6 | 6.7 | 3.1 | 2.2 | 0.5 | 7.8 | 7.9 |
| Single | 27.0 | 16.5 | 5.2 | 6.1 | 6.6 | 6.9 | 3.1 | 5.0 | 14.3 | 31.0 |
| Divorced/widowed | 18.5 | 11.2 | 53.3 | 35.5 | 2.4 | 4.0 | 26.2 | - | 62.8 | 36.7 |
| Employment/occupation | | | | | | | | | | |
| Full-time employee | 24.6 | NA | 8.9 | NA | 6.2 | NA | 4.3 | NA | 17.1 | NA |
| Self-employed | 46.7 | NA | 21.7 | NA | 8.0 | NA | 10.0 | NA | 13.9 | NA |
| Part-time employee, contractor | 21.5 | NA | 20.5 | NA | 3.5 | NA | 10.2 | NA | 23.8 | NA |
| Domestic worker | NA | 21.3 | NA | 5.9 | NA | 0.5 | NA | - | NA | 12.3 |
| Retired | NA | 16.8 | NA | 4.5 | NA | 4.1 | NA | 1.0 | NA | 2.2 |
| Student | NA | 15.7 | NA | 26.3 | NA | 6.6 | NA | 6.1 | NA | 52.3 |
| Unemployed | NA | 12.5 | NA | 3.3 | NA | 3.9 | NA | 0.8 | NA | 12.4 |
| Cumulative COVID-19 cases in residing area[g] | | | | | | | | | | |
| 1st tertile (1–12 cases per 100,000) | 27.6 | 15.2 | 4.5 | 4.3 | 6.2 | 4.8 | 2.1 | 1.6 | 9.9 | 14.8 |
| 2nd tertile (13–29 cases per 100,000) | 28.2 | 12.0 | 5.0 | 16.4 | 6.0 | 3.5 | 2.5 | 1.8 | 7.8 | 25.6 |
| 3rd tertile (30–149 cases per 100,000) | 27.2 | 15.2 | 21.7 | 5.8 | 6.0 | 4.3 | 10.6 | 2.1 | 27.4 | 15.2 |
| History of physical condition[h] | | | | | | | | | | |
| Never | 26.7 | 14.6 | 6.1 | 10.3 | 5.7 | 4.0 | 1.8 | 2.3 | 12.9 | 24.7 |
| Past | 28.5 | 12.7 | 15.5 | 3.4 | 7.9 | 5.0 | 10.9 | 1.4 | 16.5 | 9.7 |
| Present | 29.7 | 14.9 | 32.7 | 5.2 | 5.7 | 4.4 | 16.9 | 0.9 | 34.4 | 5.7 |
| History of mental condition[h] | | | | | | | | | | |
| Never | 27.6 | 13.5 | 6.1 | 8.1 | 6.2 | 4.1 | 1.9 | 1.8 | 11.8 | 17.8 |
| Past | 30.7 | 19.0 | 23.7 | 5.1 | 5.1 | 4.9 | 20.4 | 1.6 | 24.7 | 17.5 |
| Present | 24.8 | 28.2 | 54.1 | 9.6 | 5.1 | 7.5 | 27.9 | 3.6 | 56.6 | 17.0 |

COVID-19, coronavirus 2019; NA, not applicable.

The prevalence was weighted to account for selection in an internet survey.

[a] Income loss was assessed by asking respondents whether their income had decreased compared to that before the COVID-19 pandemic.

[b] Money shortage to buy things or pay bills was defined as any problems to pay for items including rent/mortgage, food, medical/dental fees, tuition, and other necessities. Percentages that reported experiencing such problems for the first time during the COVID-19 pandemic were calculated.

[c] Financial anxiety was assessed by asking respondents whether they had felt anxious about the household budget during April–September 2020.

[d] Financial exploitation was defined as an event that respondents' saving or pension was used or taken by someone including their family without their permission.

[e] Non-receipt of the cash relief ($930 across-the-board handout) was defined as a denial to the question asking whether respondents had received the relief by the time of survey.

[f] Past-year income level was classified using the 2018 national baseline income (poverty line).

[g] Cumulative COVID-19 cases during January 1st–August 25th, 2020 were calculated for each of the prefectures of respondents' residence.

[h] History of illness was assessed with aggregate variables for physical illness (any cancer or malignant tumor, diabetes, asthma, bronchitis, chronic obstructive pulmonary disorder, angina, myocardial infarction, and/or cerebral infarction) and mental illness (depression or any other mental disorder).

**eTable 3.** Prevalence and correlates of economic hardships experienced during the COVID-19 pandemic among females by working status, 2020, Japan

| | Income loss[a] | | Money shortage[b] | | Financial anxiety[c] | | Financial exploitation[d] | | Non-receipt of the cash relief[e] | |
|---|---|---|---|---|---|---|---|---|---|---|
| | Working (N=6,363) Prevalence (%) | Non-working (N=6,446) Prevalence (%) | Working (N=6,363) Prevalence (%) | Non-working (N=6,446) Prevalence (%) | Working (N=6,363) Prevalence (%) | Non-working (N=6,446) Prevalence (%) | Working (N=6,363) Prevalence (%) | Non-working (N=6,446) Prevalence (%) | Working (N=6,363) Prevalence (%) | Non-working (N=6,446) Prevalence (%) |
| Female overall | 29.3 | 22.6 | 8.9 | 6.8 | 11.9 | 8.3 | 1.6 | 0.9 | 8.4 | 10.5 |
| Age, years | | | | | | | | | | |
| 15–24 | 21.8 | 21.0 | 11.1 | 9.0 | 10.0 | 9.3 | 3.7 | 3.4 | 15.7 | 26.4 |
| 25–34 | 25.7 | 28.8 | 9.0 | 11.9 | 14.5 | 18.0 | 1.9 | 1.4 | 9.7 | 11.7 |
| 35–44 | 27.0 | 24.7 | 6.7 | 8.3 | 12.6 | 14.1 | 2.2 | - | 6.6 | 9.0 |
| 45–54 | 30.0 | 26.4 | 6.9 | 7.2 | 11.7 | 11.4 | 1.6 | 0.9 | 6.5 | 10.4 |
| 55–64 | 28.3 | 21.1 | 6.0 | 4.9 | 10.6 | 6.0 | - | - | 6.7 | 6.3 |
| ≥65 | 43.2 | 20.7 | 18.0 | 5.0 | 10.4 | 4.1 | - | 0.2 | 10.6 | 6.1 |
| Education | | | | | | | | | | |
| Junior high/high school | 28.3 | 19.6 | 7.8 | 6.7 | 12.2 | 8.2 | 1.2 | 1.2 | 8.6 | 11.7 |
| Some college | 27.2 | 23.5 | 8.0 | 7.5 | 12.4 | 9.8 | 2.1 | 0.7 | 7.1 | 9.1 |
| College or higher | 34.3 | 26.6 | 12.1 | 6.6 | 11.0 | 7.7 | 1.8 | 0.4 | 9.6 | 9.0 |
| Income level in the past year[f] | | | | | | | | | | |
| ≥Baseline income (≥twice the baseline) | 31.2 | 32.1 | 8.3 | 9.6 | 10.9 | 10.8 | 1.5 | 0.4 | 7.4 | 6.1 |
| ≥Baseline income (<twice the baseline) | 39.9 | 23.5 | 12.9 | 5.3 | 16.7 | 6.9 | 1.4 | 0.5 | 5.7 | 8.6 |
| <Baseline income | 29.5 | 28.6 | 11.9 | 12.5 | 16.1 | 13.9 | 3.5 | 1.0 | 14.4 | 13.4 |
| Indeterminate (did not answer) | 13.8 | 12.0 | 4.9 | 3.9 | 7.6 | 5.8 | 1.4 | 1.5 | 12.0 | 15.2 |
| Marital status | | | | | | | | | | |
| Married | 33.0 | 23.8 | 8.9 | 7.4 | 13.9 | 9.6 | 1.5 | 0.4 | 7.8 | 7.6 |
| Single | 25.4 | 19.8 | 10.7 | 5.6 | 9.2 | 6.5 | 2.1 | 2.3 | 11.6 | 20.8 |
| Divorced/widowed | 21.6 | 21.1 | 5.3 | 5.5 | 8.7 | 4.3 | 1.0 | 0.4 | 4.6 | 5.9 |
| Employment/occupation | | | | | | | | | | |
| Full-time employee | 22.8 | NA | 6.9 | NA | 10.9 | NA | 1.7 | NA | 8.2 | NA |
| Self-employed | 48.0 | NA | 7.4 | NA | 21.2 | NA | 1.0 | NA | 7.6 | NA |
| Part-time employee, contractor | 31.1 | NA | 10.7 | NA | 11.1 | NA | 1.6 | NA | 8.8 | NA |
| Domestic worker | NA | 22.8 | NA | 6.7 | NA | 9.1 | NA | 0.5 | NA | 6.5 |
| Retired | NA | 38.1 | NA | 9.6 | NA | 9.6 | NA | 0.0 | NA | - |
| Student | NA | 16.5 | NA | 8.0 | NA | 7.6 | NA | 3.9 | NA | 26.8 |
| Unemployed | NA | 16.1 | NA | 4.3 | NA | 5.5 | NA | 0.5 | NA | 19.4 |
| Cumulative COVID-19 cases in residing area[g] | | | | | | | | | | |
| 1st tertile (1–12 cases per 100,000) | 27.1 | 20.6 | 7.2 | 9.9 | 11.8 | 11.0 | 2.4 | 1.5 | 7.4 | 9.0 |
| 2nd tertile (13–29 cases per 100,000) | 24.2 | 24.3 | 6.8 | 5.5 | 10.3 | 7.4 | 1.2 | 0.5 | 7.2 | 14.5 |
| 3rd tertile (30–149 cases per 100,000) | 34.0 | 23.2 | 11.4 | 5.2 | 12.9 | 6.9 | 1.1 | 0.6 | 10.0 | 9.7 |
| History of physical condition[h] | | | | | | | | | | |
| Never | 27.4 | 23.1 | 7.4 | 7.1 | 11.2 | 8.5 | 1.5 | 1.0 | 7.9 | 10.9 |
| Past | 29.2 | 15.6 | 8.8 | 5.1 | 14.8 | 7.4 | 1.7 | 0.8 | 6.3 | 12.0 |
| Present | 45.4 | 31.8 | 21.8 | 7.4 | 12.1 | 8.6 | 2.5 | 0.4 | 17.2 | 5.5 |
| History of mental condition[h] | | | | | | | | | | |
| Never | 28.0 | 21.3 | 8.3 | 5.2 | 11.1 | 6.2 | 1.5 | 0.8 | 8.4 | 10.9 |
| Past | 33.4 | 19.5 | 12.9 | 10.0 | 17.6 | 15.5 | 1.7 | - | 7.6 | 4.2 |
| Present | 43.8 | 43.5 | 12.7 | 23.7 | 16.3 | 27.3 | 2.9 | 2.8 | 10.2 | 13.1 |

COVID-19, coronavirus 2019; NA, not applicable.

The prevalence was weighted to account for selection in an internet survey.

[a] Income loss was assessed by asking respondents whether their income had decreased compared to that before the COVID-19 pandemic.

[b] Money shortage to buy things or pay bills was defined as any problems to pay for items including rent/mortgage, food, medical/dental fees, tuition, and other necessities. Percentages that reported experiencing such problems for the first time during the COVID-19 pandemic were calculated.

[c] Financial anxiety was assessed by asking respondents whether they had felt anxious about the household budget during April–September 2020.

[d] Financial exploitation was defined as an event that respondents' saving or pension was used or taken by someone including their family without their permission.

[e] Non-receipt of the cash relief ($930 across-the-board handout) was defined as a denial to the question asking whether respondents had received the relief by the time of survey.

[f] Past-year income level was classified using the 2018 national baseline income (poverty line).

[g] Cumulative COVID-19 cases during January 1st–August 25th, 2020 were calculated for each of the prefectures of respondents' residence.

[h] History of illness was assessed with aggregate variables for physical illness (any cancer or malignant tumor, diabetes, asthma, bronchitis, chronic obstructive pulmonary disorder, angina, myocardial infarction, and/or cerebral infarction) and mental illness (depression or any other mental disorder).

**eTable 4.** Prevalence and correlates of deterioration of physical or mental health and current state of health during the COVID-19 pandemic among males by working status, 2020, Japan

| | Physical health deterioration[a] | | Mental health deterioration[a] | | Poor/fair self-rated health[b] | | Serious psychological distress[c] | |
|---|---|---|---|---|---|---|---|---|
| | Working (N=9,008) Prevalence (%) | Non-working (N=3,665) Prevalence (%) | Working (N=9,008) Prevalence (%) | Non-working (N=3,665) Prevalence (%) | Working (N=9,008) Prevalence (%) | Non-working (N=3665) Prevalence (%) | Working (N=9,008) Prevalence (%) | Non-working (N=3,665) Prevalence (%) |
| Male overall | 14.9 | 16.8 | 16.6 | 14.6 | 12.2 | 20.7 | 13.9 | 3.3 |
| Age, years | | | | | | | | |
| 15–24 | 39.6 | 18.8 | 40.8 | 20.7 | 20.1 | 4.8 | 75.9 | 8.5 |
| 25–34 | 18.0 | 67.0 | 13.9 | 69.5 | 13.7 | 67.8 | 24.6 | 6.1 |
| 35–44 | 13.8 | 9.7 | 17.0 | 10.8 | 8.5 | 17.5 | 8.1 | 11.3 |
| 45–54 | 11.8 | 12.1 | 15.6 | 16.1 | 11.1 | 19.3 | 6.3 | 9.1 |
| 55–64 | 12.1 | 22.5 | 13.3 | 17.6 | 12.0 | 26.5 | 2.9 | 3.3 |
| ≥65 | 7.3 | 11.4 | 10.9 | 7.3 | 14.8 | 19.7 | 2.1 | 0.7 |
| Education | | | | | | | | |
| Junior high/high school | 11.2 | 13.2 | 13.8 | 10.6 | 11.3 | 18.2 | 8.3 | 3.0 |
| Some college | 12.8 | 10.6 | 17.7 | 9.4 | 10.3 | 18.9 | 7.5 | 6.6 |
| College or higher | 19.5 | 26.6 | 19.4 | 26.3 | 13.5 | 28.1 | 21.7 | 3.8 |
| Income level in the past year[f] | | | | | | | | |
| ≥Baseline income (≥twice the baseline) | 16.5 | 13.5 | 15.3 | 12.3 | 12.6 | 16.8 | 13.3 | 3.8 |
| ≥Baseline income (<twice the baseline) | 13.7 | 16.8 | 24.5 | 8.0 | 12.7 | 19.1 | 14.8 | 2.2 |
| <Baseline income | 15.5 | 30.1 | 15.9 | 32.8 | 11.5 | 38.6 | 37.2 | 5.0 |
| Indeterminate (did not answer) | 8.4 | 9.4 | 12.6 | 10.9 | 10.0 | 12.1 | 5.8 | 3.0 |
| Marital status | | | | | | | | |
| Married | 11.3 | 12.0 | 14.5 | 8.8 | 9.6 | 18.8 | 4.9 | 1.3 |
| Single | 13.8 | 17.4 | 18.5 | 17.9 | 12.5 | 13.7 | 10.7 | 8.5 |
| Divorced/widowed | 31.5 | 40.8 | 22.9 | 36.6 | 22.9 | 47.9 | 55.0 | 1.2 |
| Employment/occupation | | | | | | | | |
| Full-time employee | 13.2 | NA | 16.9 | NA | 10.4 | NA | 10.0 | NA |
| Self-employed | 17.5 | NA | 11.8 | NA | 12.2 | NA | 22.9 | NA |
| Part-time employee, contractor | 20.0 | NA | 20.0 | NA | 20.2 | NA | 22.0 | NA |
| Domestic worker | NA | 26.8 | NA | 18.0 | NA | 23.4 | NA | 8.7 |
| Retired | NA | 14.0 | NA | 5.6 | NA | 21.2 | NA | 0.7 |
| Student | NA | 34.3 | NA | 36.3 | NA | 22.1 | NA | 6.0 |
| Unemployed | NA | 11.2 | NA | 10.4 | NA | 19.8 | NA | 3.0 |
| Cumulative COVID-19 cases in residing area[g] | | | | | | | | |
| 1st tertile (1–12 cases per 100,000) | 10.9 | 9.9 | 14.9 | 7.3 | 10.6 | 19.2 | 5.7 | 2.1 |
| 2nd tertile (13–29 cases per 100,000) | 12.8 | 27.1 | 15.7 | 26.4 | 10.1 | 27.0 | 7.0 | 3.8 |
| 3rd tertile (30–149 cases per 100,000) | 18.7 | 16.3 | 18.2 | 13.3 | 14.3 | 17.8 | 22.6 | 4.2 |
| History of physical condition[f] | | | | | | | | |
| Never | 10.1 | 17.7 | 13.9 | 17.9 | 7.1 | 16.9 | 7.7 | 3.4 |
| Past | 14.6 | 16.8 | 16.2 | 12.2 | 11.6 | 18.4 | 16.3 | 4.7 |
| Present | 31.4 | 14.6 | 25.9 | 7.8 | 29.7 | 31.9 | 32.6 | 2.3 |
| History of mental condition[f] | | | | | | | | |
| Never | 9.8 | 16.2 | 13.0 | 13.7 | 8.7 | 19.2 | 6.2 | 2.1 |
| Past | 18.5 | 20.4 | 21.6 | 19.0 | 14.2 | 21.1 | 28.1 | 6.4 |
| Present | 50.5 | 26.4 | 40.2 | 28.7 | 36.9 | 50.5 | 60.9 | 25.6 |

COVID-19, coronavirus 2019; NA, not applicable.

The prevalence was weighted to account for selection in an internet survey.

[a] Deterioration of health were self-reported by asking respondents whether their state of health had worsened or improved compared to that in or during January 2020.

[b] Unfavorable general health status was defined as responses “poor” or “fair” to the question about respondents’ current health status using a 5-point Likert scale.

[c] Presence of serious psychological distress was defined as the score ≥13 on the Kessler (K6) scale.

[d] Past-year income level was classified using the latest national baseline income (poverty line).

[e] Cumulative COVID-19 cases during January 1st–August 25th, 2020 were calculated for each of the prefectures of respondents’ residence.

[f] History of illness was assessed with aggregate variables for physical illness (any cancer or malignant tumor, diabetes, asthma, bronchitis, chronic obstructive pulmonary disorder, angina, myocardial infarction, and/or cerebral infarction) and mental illness (depression or any other mental disorder).

**eTable 5.** Prevalence and correlates of deterioration of physical or mental health and current state of health during the COVID-19 pandemic among females by working status, 2020, Japan

| | Physical health deterioration[a] | | Mental health deterioration[a] | | Poor/fair self-rated health[b] | | Serious psychological distress[c] | |
|---|---|---|---|---|---|---|---|---|
| | Working (N=6,363) Prevalence (%) | Non-working (N=6,446) Prevalence (%) | Working (N=6,363) Prevalence (%) | Non-working (N=6,446) Prevalence (%) | Working (N=6,363) Prevalence (%) | Non-working (N=6,446) Prevalence (%) | Working (N=6,363) Prevalence (%) | Non-working (N=6,446) Prevalence (%) |
| Male overall | 16.4 | 14.6 | 21.8 | 20.1 | 11.4 | 13.6 | 9.5 | 8.5 |
| Age, years | | | | | | | | |
| 15–24 | 15.4 | 16.1 | 27.1 | 32.0 | 8.8 | 9.2 | 16.8 | 13.5 |
| 25–34 | 19.1 | 19.3 | 27.4 | 27.1 | 8.9 | 9.5 | 13.5 | 11.2 |
| 35–44 | 13.9 | 19.7 | 20.1 | 22.3 | 10.7 | 15.1 | 8.8 | 13.6 |
| 45–54 | 15.7 | 14.0 | 20.6 | 20.2 | 11.3 | 16.8 | 7.3 | 9.8 |
| 55–64 | 19.4 | 15.2 | 21.7 | 20.2 | 10.0 | 16.6 | 5.3 | 6.8 |
| ≥65 | 15.5 | 11.5 | 15.6 | 13.2 | 20.1 | 13.4 | 9.5 | 4.8 |
| Education | | | | | | | | |
| Junior high/high school | 16.4 | 16.1 | 21.7 | 21.7 | 11.4 | 15.2 | 10.1 | 8.2 |
| Some college | 15.4 | 16.7 | 21.7 | 21.2 | 8.5 | 12.8 | 7.7 | 10.3 |
| College or higher | 17.0 | 10.4 | 21.1 | 16.6 | 14.9 | 11.0 | 11.0 | 7.3 |
| Income level in the past year[f] | | | | | | | | |
| ≥Baseline income (≥twice the baseline) | 15.0 | 16.6 | 19.9 | 20.1 | 8.3 | 13.0 | 6.4 | 11.4 |
| ≥Baseline income (<twice the baseline) | 20.8 | 15.7 | 26.2 | 16.2 | 18.5 | 12.4 | 14.6 | 6.3 |
| <Baseline income | 19.3 | 19.2 | 25.0 | 19.6 | 16.0 | 20.2 | 15.2 | 13.8 |
| Indeterminate (did not answer) | 13.8 | 11.0 | 20.0 | 23.0 | 9.2 | 13.4 | 9.0 | 6.3 |
| Marital status | | | | | | | | |
| Married | 15.9 | 14.8 | 20.0 | 19.3 | 9.9 | 14.3 | 8.0 | 7.7 |
| Single | 16.8 | 14.6 | 26.6 | 25.0 | 14.2 | 11.9 | 13.5 | 12.4 |
| Divorced/widowed | 17.8 | 13.5 | 19.4 | 13.8 | 12.4 | 12.7 | 7.9 | 5.1 |
| Employment/occupation | | | | | | | | |
| Full-time employee | 17.0 | NA | 24.0 | NA | 9.9 | NA | 9.8 | NA |
| Self-employed | 24.8 | NA | 23.7 | NA | 19.1 | NA | 18.5 | NA |
| Part-time employee, contractor | 14.6 | NA | 19.7 | NA | 11.3 | NA | 7.9 | NA |
| Domestic worker | NA | 15.0 | NA | 18.1 | NA | 12.6 | NA | 6.9 |
| Retired | NA | 12.3 | NA | 12.2 | NA | 12.1 | NA | 9.3 |
| Student | NA | 13.6 | NA | 31.6 | NA | 8.1 | NA | 8.7 |
| Unemployed | NA | 15.6 | NA | 24.0 | NA | 21.9 | NA | 13.6 |
| Cumulative COVID-19 cases in residing area[g] | | | | | | | | |
| 1st tertile (1–12 cases per 100,000) | 16.3 | 17.5 | 21.7 | 22.7 | 10.8 | 17.1 | 10.3 | 11.5 |
| 2nd tertile (13–29 cases per 100,000) | 13.9 | 15.0 | 21.4 | 19.4 | 9.1 | 10.7 | 7.6 | 8.8 |
| 3rd tertile (30–149 cases per 100,000) | 17.8 | 12.5 | 22.0 | 18.7 | 13.3 | 12.6 | 10.0 | 6.4 |
| History of physical condition[f] | | | | | | | | |
| Never | 15.6 | 14.1 | 21.5 | 19.6 | 10.7 | 10.7 | 9.1 | 8.4 |
| Past | 19.4 | 13.7 | 23.7 | 18.0 | 12.1 | 13.4 | 9.9 | 7.8 |
| Present | 16.8 | 19.6 | 19.8 | 27.6 | 16.4 | 33.3 | 12.8 | 10.7 |
| History of mental condition[f] | | | | | | | | |
| Never | 14.7 | 12.1 | 19.6 | 17.3 | 9.8 | 10.5 | 7.2 | 5.1 |
| Past | 25.6 | 18.4 | 35.4 | 22.8 | 15.5 | 15.2 | 19.2 | 13.7 |
| Present | 28.2 | 44.0 | 35.3 | 54.0 | 29.6 | 52.6 | 30.4 | 48.0 |

COVID-19, coronavirus 2019; NA, not applicable.

The prevalence was weighted to account for selection in an internet survey. See also **eTable 1** for detailed definitions of economic hardship measures.

[a] Deterioration of health were self-reported by asking respondents whether their state of health had worsened or improved compared to that in or during January 2020.

[b] Unfavorable general health status was defined as responses “poor” or “fair” to the question about respondents’ current health status using a 5-point Likert scale.

[c] Presence of serious psychological distress was defined as the score ≥13 on the Kessler (K6) scale.

[d] Past-year income level was classified using the latest national baseline income (poverty line).

[e] Cumulative COVID-19 cases during January 1st–August 25th, 2020 were calculated for each of the prefectures of respondents’ residence.

[f] History of illness was assessed with aggregate variables for physical illness (any cancer or malignant tumor, diabetes, asthma, bronchitis, chronic obstructive pulmonary disorder, angina, myocardial infarction, and/or cerebral infarction) and mental illness (depression or any other mental disorder).

**eTable 6.** Associations between economic hardships and self-reported health during the COVID-19 pandemic, 2020, Japan

| Economic hardships/population subgroups | Deterioration of physical health[a] APR (95% CI) | Deterioration of mental health[a] APR (95% CI) | Unfavorable self-rated health condition[b] APR (95% CI) | Presence of serious psychological distress[c] APR (95% CI) |
|---|---|---|---|---|
| Income loss | | | | |
| Overall | 1.68 (1.43–1.98) | 1.62 (1.36–1.93) | 1.28 (1.01–1.61) | 1.64 (1.28–2.10) |
| Male, working | 1.95 (1.52–2.50) | 1.63 (1.12–2.37) | 1.06 (0.71–1.58) | 1.41 (1.11–1.79) |
| Male, non-working | 1.45 (1.01–2.06) | 1.60 (1.17–2.18) | 1.15 (0.80–1.67) | 1.87 (1.06–3.27) |
| Female, working | 1.59 (1.28–1.98) | 1.68 (1.40–2.00) | 1.57 (1.22–2.03) | 1.62 (1.22–2.14) |
| Female, non-working | 1.55 (1.20–2.00) | 1.47 (1.12–1.92) | 1.36 (1.00–1.85) | 2.01 (1.50–2.69) |
| Money shortage to buy things/pay bills (experienced first time) | | | | |
| Overall | 1.75 (1.18–2.60) | 2.07 (1.60–2.68) | 1.79 (1.02–3.13) | 4.14 (3.12–5.49) |
| Male, working | 1.03 (0.54–1.96) | 2.07 (1.51–2.83) | 0.66 (0.33–1.33) | 5.18 (3.72–7.21) |
| Male, non-working | 1.71 (1.17–2.49) | 2.34 (1.58–3.46) | 1.94 (1.43–2.63) | 1.60 (0.89–2.87) |
| Female, working | 1.48 (1.05–2.07) | 1.48 (1.11–1.98) | 2.53 (1.69–3.79) | 2.32 (1.59–3.37) |
| Female, non-working | 2.79 (2.09–3.72) | 2.17 (1.60–2.94) | 2.11 (1.49–2.98) | 3.59 (2.58–4.99) |
| Money shortage to buy things/pay bills (since before the COVID-19 pandemic) | | | | |
| Overall | 1.68 (1.31–2.16) | 1.88 (1.51–2.34) | 1.99 (1.50–2.63) | 2.99 (2.09–4.26) |
| Male, working | 1.78 (1.39–2.29) | 1.78 (1.40–2.27) | 1.42 (1.07–1.88) | 2.86 (2.09–3.90) |
| Male, non-working | 1.11 (0.74–1.67) | 1.55 (1.05–2.29) | 1.71 (1.26–2.33) | 1.95 (1.14–3.31) |
| Female, working | 1.99 (1.35–2.92) | 1.86 (1.32–2.61) | 2.70 (1.87–3.90) | 3.06 (1.87–5.01) |
| Female, non-working | 1.48 (1.11–1.96) | 1.80 (1.25–2.61) | 2.00 (1.36–2.95) | 2.71 (1.87–3.92) |
| Anxiety about finance (experienced first time) | | | | |
| Overall | 2.52 (2.00–3.18) | 2.84 (2.37–3.39) | 1.87 (1.33–2.62) | 3.52 (2.44–5.08) |
| Male, working | 2.17 (1.74–2.71) | 3.10 (2.47–3.89) | 1.34 (1.01–1.76) | 2.36 (1.78–3.14) |
| Male, non-working | 2.56 (1.67–3.93) | 3.53 (2.46–5.06) | 1.86 (1.25–2.76) | 3.61 (2.21–5.92) |
| Female, working | 2.58 (1.83–3.64) | 2.78 (2.12–3.64) | 2.34 (1.52–3.62) | 3.82 (2.40–6.10) |
| Female, non-working | 3.29 (2.51–4.31) | 2.61 (2.02–3.37) | 2.09 (1.44–3.03) | 4.44 (3.21–6.14) |
| Anxiety about finance (since before the COVID-19 pandemic) | | | | |
| Overall | 1.47 (1.07–2.02) | 1.65 (1.27–2.14) | 1.20 (0.81–1.78) | 2.69 (1.85–3.92) |
| Male, working | 1.09 (0.70–1.71) | 1.55 (0.85–2.83) | 0.54 (0.27–1.05) | 2.09 (1.38–3.17) |
| Male, non-working | 1.95 (1.19–3.19) | 2.2 (1.47–3.3) | 2.48 (1.81–3.39) | 4.49 (2.39–8.40) |
| Female, working | 2.01 (1.59–2.54) | 2.04 (1.68–2.46) | 1.71 (1.24–2.37) | 2.47 (1.83–3.33) |
| Female, non-working | 2.24 (1.71–2.92) | 1.59 (1.24–2.04) | 2.18 (1.68–2.81) | 3.47 (2.43–4.94) |
| Financial exploitation | | | | |
| Overall | 1.13 (0.86–2.64) | 0.63 (0.27–1.47) | 0.71 (0.20–2.49) | 1.99 (1.18–3.37) |
| Male, working | 1.12 (0.59–2.13) | 0.48 (0.20–1.12) | 1.10 (0.59–2.05) | 2.07 (1.16–3.72) |
| Male, non-working | 1.88 (1.01–3.48) | 1.80 (1.00–3.26) | 1.52 (0.96–2.42) | 5.04 (2.53–10.07) |
| Female, working | 1.21 (0.82–1.80) | 1.19 (0.82–1.71) | 1.57 (0.99–2.50) | 3.24 (2.34–4.49) |
| Female, non-working | 1.33 (0.67–2.62) | 2.23 (1.34–3.72) | 0.80 (0.43–1.47) | 2.10 (1.24–3.55) |
| Non-receipt of the government subsidy | | | | |
| Overall | 0.78 (0.54–1.11) | 0.94 (0.69–1.28) | 1.31 (0.95–1.80) | 1.28 (0.93–1.76) |
| Male, working | 0.61 (0.38–1.00) | 0.97 (0.52–1.80) | 1.33 (0.75–2.34) | 1.28 (0.95–1.71) |
| Male, non-working | 0.72 (0.45–1.16) | 1.06 (0.59–1.88) | 1.44 (0.91–2.27) | 0.72 (0.31–1.66) |
| Female, working | 1.02 (0.73–1.41) | 0.76 (0.57–1.01) | 1.32 (0.88–1.98) | 1.14 (0.81–1.60) |
| Female, non-working | 0.66 (0.45–0.98) | 0.86 (0.59–1.24) | 0.97 (0.66–1.44) | 0.92 (0.62–1.36) |

APR, adjusted prevalence ratio; CI, confidence interval; COVID-19, coronavirus 2019.

N=9,008 (male, working); 3,665 (male, non-working); 6,446 (female, working); 6,363 (female, non-working). APRs and 95% CIs estimated through weighted multivariable-adjusted log-linear models followed by robust variance estimation controlling for working status (for overall only), age, education, past-year income level, marital status, cumulative COVID-19 cases in residing area, and history of physical and mental illness. Working status was categorized as to be working (full-time employee, self-employed, part-time/contractor) or non-working (retired, student, domestic worker, unemployed).

[a] Deterioration of health were self-reported by asking respondents whether their state of health had worsened or improved compared to that in or during January 2020.

[b] Unfavorable general health status was defined as responses “poor” or “fair” to the question about respondents’ current health status using a 5-point Likert scale.

[c] Presence of serious psychological distress was defined as the score ≥13 on the Kessler (K6) scale.

**eTable 7.** Associations between economic hardships and self-reported health during the COVID-19 pandemic by age (<65 vs. ≥65 years), 2020, Japan

| Economic hardships/population subgroups | Age <65 years old | | | | Age ≥65 years old | | | |
|---|---|---|---|---|---|---|---|---|
| | Deterioration of physical health | Deterioration of mental health | Unfavorable self-rated health condition | Presence of serious psychological distress | Deterioration of physical health | Deterioration of mental health | Unfavorable self-rated health condition | Presence of serious psychological distress |
| | APR (95% CI) | APR (95% CI) | APR (95% CI) | APR (95% CI) | APR (95% CI) | APR (95% CI) | APR (95% CI) | APR (95% CI) |
| Income loss | | | | | | | | |
| Overall | 1.68 (1.45–1.94) | 1.59 (1.34–1.88) | 1.17 (0.95–1.44) | 1.42 (1.18–1.70) | 1.80 (1.17–2.77) | 1.68 (0.99–2.85) | 1.59 (1.04–2.44) | 5.03 (2.52–10.02) |
| Male, working | 1.89 (1.49–2.41) | 1.58 (1.07–2.35) | 1.05 (0.67–1.64) | 1.38 (1.09–1.74) | 1.44 (0.72–2.89) | 1.57 (0.90–2.74) | 1.06 (0.57–1.98) | 1.59 (0.61–4.12) |
| Male, non-working | 1.48 (0.95–2.30) | 1.68 (1.15–2.45) | 0.96 (0.71–1.31) | 1.71 (0.95–3.09) | 1.45 (0.92–2.28) | 1.52 (0.89–2.59) | 1.40 (0.90–2.19) | 4.11 (1.40–12.1) |
| Female, working | 1.56 (1.33–1.85) | 1.68 (1.47–1.92) | 1.31 (1.07–1.60) | 1.52 (1.23–1.89) | 1.98 (1.11–3.54) | 1.67 (0.90–3.07) | 1.60 (0.73–3.53) | 0.60 (0.23–1.58) |
| Female, non-working | 1.49 (1.20–1.87) | 1.53 (1.27–1.85) | 1.38 (1.11–1.72) | 1.67 (1.25–2.25) | 1.78 (1.17–2.72) | 1.41 (0.71–2.82) | 1.41 (0.75–2.64) | 4.06 (1.97–8.37) |
| Money shortage to buy things/pay bills (experienced first time) | | | | | | | | |
| Overall | 1.65 (1.10–2.47) | 1.99 (1.57–2.52) | 1.33 (0.73–2.42) | 3.68 (2.80–4.83) | 2.55 (1.47–4.43) | 2.96 (1.60–5.46) | 3.29 (1.81–6.00) | 7.83 (4.00–15.33) |
| Male, working | 1.13 (0.63–2.01) | 2.06 (1.51–2.80) | 0.61 (0.29–1.29) | 5.47 (3.97–7.53) | 2.22 (0.73–6.73) | 4.60 (2.13–9.95) | 1.53 (0.58–4.02) | 5.58 (1.66–18.73) |
| Male, non-working | 1.97 (1.26–3.08) | 3.00 (1.96–4.57) | 2.02 (1.39–2.93) | 1.59 (0.86–2.95) | 0.99 (0.47–2.07) | 1.08 (0.51–2.29) | 1.47 (0.85–2.55) | 1.39 (0.24–7.97) |
| Female, working | 1.75 (1.41–2.17) | 1.73 (1.43–2.09) | 1.85 (1.41–2.44) | 2.58 (1.98–3.36) | 1.02 (0.14–7.34) | 0.31 (0.03–3.86) | 2.01 (0.97–4.17) | – |
| Female, non-working | 2.09 (1.59–2.74) | 1.53 (1.19–1.97) | 1.43 (1.07–1.91) | 2.57 (1.82–3.61) | 5.05 (3.44–7.41) | 5.90 (3.97–8.76) | 3.16 (1.82–5.48) | 8.15 (3.72–17.87) |
| Money shortage to buy things/pay bills (since before the COVID-19 pandemic) | | | | | | | | |
| Overall | 1.57 (1.34–1.85) | 1.59 (1.39–1.82) | 1.63 (1.37–1.94) | 2.50 (2.01–3.09) | 2.32 (1.05–5.11) | 3.92 (2.20–6.97) | 2.98 (1.82–4.88) | 7.58 (3.73–15.4) |
| Male, working | 1.72 (1.35–2.19) | 1.70 (1.35–2.15) | 1.38 (1.08–1.76) | 2.68 (1.94–3.70) | 1.09 (0.48–2.46) | 2.13 (1.07–4.22) | 1.51 (0.63–3.61) | 2.89 (0.98–8.55) |
| Male, non-working | 1.15 (0.68–1.95) | 1.87 (1.20–2.94) | 2.28 (1.61–3.25) | 2.05 (1.24–3.38) | 1.17 (0.69–1.99) | 1.16 (0.54–2.49) | 1.65 (1.06–2.58) | 2.2 (0.38–12.82) |
| Female, working | 1.54 (1.24–1.91) | 1.50 (1.25–1.80) | 1.84 (1.45–2.33) | 2.02 (1.57–2.62) | 3.69 (2.10–6.49) | 4.66 (2.52–8.61) | 3.89 (1.96–7.72) | 5.91 (2.61–13.4) |
| Female, non-working | 1.57 (1.18–2.10) | 1.42 (1.08–1.87) | 1.45 (1.10–1.91) | 2.78 (1.96–3.94) | 1.44 (0.87–2.38) | 3.62 (2.16–6.07) | 2.88 (1.76–4.71) | 0.59 (0.15–2.26) |
| Anxiety about finance (experienced first time) | | | | | | | | |
| Overall | 2.02 (1.74–2.35) | 2.41 (2.10–2.76) | 1.35 (1.13–1.63) | 2.57 (2.10–3.16) | 7.11 (4.40–11.5) | 7.95 (5.46–11.56) | 4.28 (2.31–7.92) | 23.67 (12.2–45.92) |
| Male, working | 2.20 (1.77–2.75) | 2.89 (2.30–3.62) | 1.50 (1.16–1.94) | 2.37 (1.78–3.16) | 1.25 (0.31–5.04) | 5.92 (2.99–11.74) | 0.50 (0.09–2.65) | 3.07 (0.73–12.79) |
| Male, non-working | 2.23 (1.30–3.84) | 3.17 (2.09–4.80) | 1.78 (1.13–2.79) | 3.65 (2.17–6.14) | 3.57 (1.78–7.19) | 4.59 (2.40–8.80) | 2.15 (1.23–3.76) | 2.74 (0.77–9.75) |
| Female, working | 1.97 (1.61–2.41) | 2.31 (1.97–2.71) | 1.55 (1.21–1.99) | 2.48 (1.90–3.24) | 14.15 (6.79–29.5) | 14.35 (6.96–29.6) | 7.22 (2.5–20.87) | 41.87 (8.44–207.73) |
| Female, non-working | 2.51 (1.94–3.25) | 2.23 (1.81–2.75) | 1.45 (1.08–1.94) | 3.40 (2.43–4.75) | 5.72 (3.83–8.55) | 5.46 (3.47–8.58) | 3.73 (2.22–6.27) | 7.36 (3.31–16.39) |
| Anxiety about finance (since before the COVID-19 pandemic) | | | | | | | | |
| Overall | 1.29 (0.92–1.81) | 1.60 (1.21–2.12) | 0.95 (0.61–1.50) | 2.46 (1.72–3.52) | 2.59 (1.67–4.02) | 1.65 (1.01–2.70) | 2.09 (1.38–3.15) | 5.13 (2.24–11.76) |
| Male, working | 1.06 (0.68–1.64) | 1.49 (0.80–2.76) | 0.50 (0.25–1.00) | 2.09 (1.37–3.20) | 2.89 (1.32–6.32) | 2.43 (1.23–4.80) | 1.29 (0.59–2.86) | 2.68 (1.05–6.84) |
| Male, non-working | 1.36 (0.80–2.32) | 2.33 (1.43–3.79) | 2.12 (1.55–2.91) | 5.05 (2.52–10.11) | 2.93 (1.66–5.15) | 1.97 (0.96–4.07) | 2.76 (1.89–4.05) | 2.21 (0.50–9.74) |
| Female, working | 2.06 (1.70–2.50) | 2.05 (1.74–2.41) | 1.83 (1.46–2.30) | 2.17 (1.66–2.84) | 1.23 (0.52–2.90) | 1.73 (0.72–4.21) | 1.54 (0.57–4.13) | 34.56 (6.64–179.82) |
| Female, non-working | 1.95 (1.49–2.55) | 1.63 (1.28–2.08) | 1.88 (1.47–2.39) | 3.37 (2.39–4.76) | 2.36 (1.30–4.28) | 1.21 (0.70–2.08) | 3.17 (1.96–5.14) | 1.64 (0.60–4.48) |

| | | | | | | | | |
|---|---|---|---|---|---|---|---|---|
| Financial exploitation | | | | | | | | |
| Overall | 0.83 (0.35–1.98) | 0.61 (0.26–1.43) | 0.65 (0.19–2.27) | 1.82 (1.10–3.02) | 1.89 (0.87–4.15) | 2.43 (1.04–5.68) | 1.65 (0.85–3.22) | 3.14 (1.20–8.22) |
| Male, working | 1.32 (0.77–2.25) | 0.50 (0.22–1.12) | 1.18 (0.63–2.21) | 2.28 (1.26–4.10) | 0.26 (0.04–1.91) | 2.36 (0.83–6.70) | 1.42 (0.30–6.67) | 9.45 (1.8–49.71) |
| Male, non-working | 1.77 (0.89–3.51) | 1.55 (0.80–2.98) | 1.27 (0.64–2.54) | 4.59 (2.24–9.38) | 3.95 (2.09–7.46) | 4.54 (2.33–8.85) | 2.33 (1.15–4.70) | 5.76 (1.43–23.17) |
| Female, working | 1.14 (0.77–1.69) | 1.14 (0.79–1.65) | 1.40 (0.89–2.21) | 3.16 (2.29–4.35) | 8.24 (2.84–23.88) | 4.54 (0.69–29.86) | 4.47 (1.27–15.67) | 15.97 (2.44–104.63) |
| Female, non-working | 1.41 (0.69–2.90) | 2.40 (1.48–3.89) | 0.87 (0.45–1.72) | 2.09 (1.13–3.86) | 0.61 (0.12–3.11) | 0.52 (0.09–3.13) | 0.44 (0.11–1.72) | 1.06 (0.42–2.67) |
| Non-receipt of the government subsidy | | | | | | | | |
| Overall | 0.78 (0.55–1.11) | 0.94 (0.69–1.29) | 1.38 (1.01–1.89) | 1.22 (0.90–1.65) | 0.56 (0.26–1.21) | 0.98 (0.40–2.43) | 0.94 (0.40–2.21) | 0.43 (0.12–1.55) |
| Male, working | 0.65 (0.42–1.01) | 1.02 (0.55–1.88) | 1.39 (0.80–2.43) | 1.29 (0.96–1.75) | 0.74 (0.09–5.92) | – | 0.96 (0.31–2.99) | 6.18 (2.49–15.34) |
| Male, non-working | 0.58 (0.35–0.97) | 0.65 (0.36–1.20) | 0.81 (0.57–1.15) | 0.79 (0.35–1.82) | 1.23 (0.48–3.11) | 3.88 (1.84–8.22) | 2.39 (1.26–4.52) | – |
| Female, working | 1.11 (0.85–1.44) | 0.82 (0.63–1.06) | 1.69 (1.30–2.20) | 1.19 (0.87–1.62) | 0.99 (0.40–2.48) | 0.51 (0.11–2.27) | 0.65 (0.11–3.93) | 5.93 (1.02–34.38) |
| Female, non-working | 0.71 (0.47–1.07) | 0.89 (0.62–1.30) | 1.18 (0.82–1.69) | 0.91 (0.59–1.39) | 0.35 (0.15–0.80) | 0.57 (0.22–1.47) | 0.33 (0.11–1.02) | 0.84 (0.26–2.70) |

APR, adjusted prevalence ratio; CI, confidence interval; COVID-19, coronavirus 2019.

N=8,020 (male, working, age <65 years); 1,603 (male, non-working, age <65 years); 5,868 (female, working, age <65 years); 3,839 (female, non-working, age <65 years); N=988 (male, working, age ≥65 years); 2,062 (male, non-working, age ≥65 years); 578 (female, working, age ≥65 years); 2,524 (female, non-working, age ≥65 years). APRs and 95% CIs estimated through weighted multivariable-adjusted log-linear models followed by robust variance estimation controlling for working status (for overall only), age, education, past-year income level, marital status, cumulative COVID-19 cases in residing area, and history of physical and mental illness. Working status was categorized as to be working (full-time employee, self-employed, part-time/contractor) or non-working (retired, student, domestic worker, unemployed).

[a] Deterioration of health were self-reported by asking respondents whether their state of health had worsened or improved compared to that in or during January 2020.

[b] Unfavorable general health status was defined as responses “poor” or “fair” to the question about respondents’ current health status using a 5-point Likert scale.

[c] Presence of serious psychological distress was defined as the score ≥13 on the Kessler (K6) scale.
